# The Validity and Reliability of the Turkish Version of the Autism Family Experience Questionnaire (AFEQ)

**DOI:** 10.1007/s10803-024-06264-y

**Published:** 2024-03-08

**Authors:** Damla Eyuboglu, Murat Eyuboglu, Ferhat Yaylaci, Baris Guller, Begum Sahbudak, Aslihan Avunduk, Onur Oktay Dagli, Seval Caliskan Pala, Didem Arslantas

**Affiliations:** 1 Department of Child and Adolescent Psychiatry, Faculty of Medicine, Eskisehir Osmangazi University, 26480 Eskisehir, Turkey; 2 Department of Child and Adolescent Psychiatry, Bursa Dortcelik Children Hospital, Bursa, Turkey; 3 Child and Adolescent Psychiatry, Manisa Mental Health and Diseases Hospital, Manisa, Turkey; 4 Department of Public Health, Faculty of Medicine, Eskisehir Osmangazi University, Eskisehir, Turkey; 5 Eskisehir Provincial Health Directorate, Odunpazari Health Directorate, Eskisehir, Turkey

**Keywords:** Autism spectrum disorder, Family experience, Quality of life, Parent, Wellbeing

## Abstract

The aim of this study was to examine the reliability and validity of the Turkish version of the AFEQ for Turkish parents of children with ASD. The Turkish-translated version of the AFEQ was administered to 241 parents of children aged 2–12 years with ASD to examine the construct validity and internal consistencies. Parents completed the Autism Behavior Checklist (ABC), and Quality of Life in Autism Questionnaire Parent version, along with the AFEQ. The mean age of the children of 241 individuals in the study group was 7.63 ± 3.02 and 88.4% (*n* = 213) were male. Cronbach’s alpha coefficient was 0.921 of the total variance. Cronbach alpha coefficients are 0.813 for the “Experience of being a parent” subscale, 0.768 for the “Family Life” subscale, 0.810 for the “Child Development, Understanding and Social Relationships” subscale, and 0.804 for the “Child Symptoms (Feelings and Behaviour)” subscale. In conclusion, the translated and culturally adapted AFEQ shows good reliability and validity to measure the priorities of autistic children and their families in Turkey. It can also be useful in monitoring the effectiveness of intervention programs and changes in the child.

## Introduction

Autism Spectrum Disorder (ASD) is a neurodevelopmental condition estimated to impact one in 44 children in the USA (Maenner et al., 2021). ASD is characterized by persistent deficits in social communication and interaction across various contexts, along with restricted and repetitive patterns of behavior, interests, or activities (American Psychiatric Association [APA], 2013). ASD presents lifelong challenges in social, emotional, and behavioral domains. Despite the significance of early ASD diagnosis and appropriate interventions, delays in diagnosis and intervention often occur due to factors such as parental unawareness and limited diagnostic tools and support resources (Park et al., 2018). Timely ASD diagnosis enables access to specialized services, education, and early interventions, which have been shown to mitigate long-term cognitive, behavioral, and functional difficulties in children and enhance family outcomes (Howlin et al., 2009). Given the rising prevalence of autism diagnoses and its potential long-term implications, evaluating the family context and immediate environment becomes paramount when addressing autism.

Autism is a lifelong neurodevelopmental condition marked by strengths (de Schipper, 2016; Kirchner, 2016; Meilleur et al., 2015) in attention, memory, visuospatial abilities, and sensory processing (Baron-Cohen et al., 2009; Lee, 2023). Moreover, the ability of individuals with autism to hyper-focus on their interests can lead to expertise in areas like technology, mathematics, and art (de Schipper, 2016). Nonetheless, they may also experience challenging symptoms such as stereotypic behaviors, obsessive tendencies, meltdowns, stimming behaviors, and peer bullying. Parents play a pivotal role in safeguarding their children’s rights and interpreting their expressions, emotions, and thoughts (Morris et al., 2014). Throughout their children’s clinical journeys and development, parents provide guidance on individual and parental needs, necessary interventions, and the efficacy of implemented methods (Leadbitter et al., 2018).

While numerous studies focus on diagnosing developmental characteristics, special needs, and the quality of life of children with neurodevelopmental disorders, these domains are often examined independently (Harris et al., 2014; Skaletski et al., 2021; Sturner et al., 2023).

Quality of life encompasses multiple dimensions and encompasses functionality across various life aspects, based on perceptions of health, including physical, social, and psychological factors (Bakas et al., 2012). According to the conceptual model by Schalock and Alonso regarding the quality of life of individuals with intellectual disabilities, quality of life encompasses emotional well-being, personal development, interpersonal relationships, social inclusion, physical well-being, self-determination, material well-being, and rights (Schalock et al., 2002). Health-Related Quality of Life (HRQoL) pertains to the impact of an individual’s current illness on psychosocial, mental, and physical well-being (Fayers et al., 2013). Commonly used tools for assessing children’s quality of life include the Pediatric Quality of Life Inventory and the Pediatric Quality of Life Questionnaire (PedsQL) (Ikeda et al., 2014; Varni et al., 1999). Similarly, tools such as the Quality of Life in Autism Questionnaire-Parent Version and the Family Quality of Life Scale assess the quality of life of families of children with autism (Eapen et al., 2014; Hofman et al., 2006).

Various tools, such as the Social Responsiveness Scale, Autism Spectrum Rating Scales, Aberrant Behavior Checklist (ABC), Child Behavior Checklist, Autism Spectrum Rating Scales (ASRS), Childhood Autism Rating Scale, and Social Communication Questionnaire, are used to screen and evaluate autism and related symptoms (Achenbach et al., 1991; Aman et al., 1985; Constantino et al., 2012; Goldstein et al., 2009; Rutter et al., 2003; Schopler et al., 2010). Despite this assortment of tools, instruments that comprehensively assess both the child and the family together are limited.

The Autism Family Experience Questionnaire (AFEQ) was developed by Leadbitter et al. (2018) to evaluate parents’ personal and family experiences, as well as the developmental and emotional-behavioral expressions of their autistic children. The AFEQ’s subscales facilitate joint assessment by autistic children and their parents of their autism-related experiences on both individual and family levels. Additionally, the questionnaire was designed to appraise the effectiveness of emerging autism intervention programs and was employed to evaluate the impact of Pre-school Autism Communication Therapy (PACT Therapy), as discussed by the authors. PACT Therapy entails parent-mediated video-assisted communication-focused intervention for preschool children with autism and their parents (Leadbitter et al., 2018). This intervention targets social interactive and communication impairments in autism, aiming to enhance child-parent communication by developing parental strategies to address the social and communicative challenges faced by children with autism (Green et al., 2010).

The authors developed the AFEQ recognizing the importance of assessing the effects of autism intervention programs from multiple perspectives, encompassing their impact on both the child and the family (Leadbitter et al., 2018). They asserted that this assessment tool effectively reflects family experiences, quality of life, and priorities. The questionnaire underwent validation using a sample of autistic children and their parents across three UK centers, demonstrating favorable internal consistency and convergent validity (Leadbitter et al., 2018).

To the best of our knowledge, only the English version of this questionnaire is available. Thus, the present study aims to explore the reliability and validity of the Turkish version of the AFEQ among Turkish parents of children with ASD.

## Methods

### Data Collection

The study participants comprised 277 children aged 2 to 12 years who had been diagnosed with Autism Spectrum Disorder (ASD) according to DSM-5 criteria by a Child and Adolescent Psychiatrist at the Child and Adolescent Psychiatry Outpatient Clinic of Eskişehir Osmangazi University Hospital, Eskişehir, Turkey. Thirty-six participants were excluded due to incomplete questionnaires, resulting in a total of 241 participants who completed the study. In our investigation, parents completed the Autism Family Experience Questionnaire (AFEQ), the Autism Behavior Checklist (ABC), and the Quality of Life in Autism Questionnaire Parent Version. The ages of the parents ranged from 29 to 51 years and the mean (SD) was 36.4 (7.9). 84.6% (n:204) of the parents participating in the study were mothers.

### Instruments

#### Autism Behavior Checklist (ABC)

Developed by Krug and colleagues (Krug, 1980), this checklist consists of 57 items divided into five subscales: sensory, relationship building, body and object use, language skills, and social skills. The minimum score is 0, and the maximum score is 159 (Krug, 1993). Yılmaz Irmak et al. conducted the Turkish validity and reliability study for children, establishing a cutoff score of 39 to identify children with suspected autism (Yılmaz Irmak et al., 2007).

#### Quality of Life in Autism Questionnaire Parent Version

This Likert-type questionnaire, developed by Eapen et al., consists of two subscales (A and B) with a five-point scale ranging from “never” to “very much.” Part A contains 28 questions concerning parental perceptions of their own quality of life, while Part B contains 20 questions addressing how autism-specific characteristics of their children create challenges for parents. Scores range from 48 to 240 (Eapen et al., 2014). The Turkish version of the Quality of Life in Autism Questionnaire was well validated and exhibited demonstrated excellent internal consistency (α = 0.93 for part A, α = 0.94 for part B**)** (Ozgur et al., 2017).

#### Autism Family Experience Questionnaire (AFEQ)

The Autism Family Experience Questionnaire, developed by Leadbitter et al. consists of 4 subscales which include the experience of being a parent of a child with autism spectrum disorder (13 items), family life (9 items), the child development, understanding, and social relations-(14 items), the child symptoms-feelings and behavior-(12 items) and includes a total of 48 items. The AFEQ features both positive and negative statements and employs a five-point ordinal scale (1 = always to 5 = never), including an “Not Applicable” option. Higher scores indicate more negative experiences. Cronbach’s alpha demonstrated high reliability for parent (α = 0.85), family (0.83), child development (0.81), child symptoms (0.79), and AFEQ total (0.92) domains (Leadbitter et al., 2018).

#### Translation of Autism Family Experience Questionnaire (AFEQ) into Turkish

Authorization to adapt the AFEQ into Turkish was obtained from the original authors. The translation adhered to the back-translation method, preserving the conceptual integrity of the scales. Four child psychiatrists conducted initial translations into Turkish, considering item suitability, validity, and cultural appropriateness. Back-translation was performed by two experts, incorporating adjustments and confirmed by the original author of AFEQ.

### Statistical Analysis

Scale factor analysis indicated Kaiser–Meyer–Olkin: 0.86 and Bartlett’s test: *p* < .001. Exploratory factor analysis (EFA) assessed construct validity. Cronbach’s alpha coefficient evaluated internal consistency. R studio software assessed model compatibility for confirmatory factor analysis, using fit indices like Chi-square/df, Root Mean Squared Approximation Error (RMSEA), Standardized Root Residual Square Mean (SRMR), and Parsimony Normed Fit Index (PNFI). Criterion validity was determined by using the ABC and Quality of Life in Autism Questionnaire. All analyses retained the full questionnaire. The Turkish AFEQ contained four domains and 48 items, each scored from 1 (always) to 5 (never). Data analysis utilized SPSS 15.0 and R studio. It was reported using descriptive statistics of the study group (frequencies, ratios, means, median) and measures of distribution (standard deviation, minimum-maximum). The Kolmogorov–Smirnov test was used to assess whether the total scale scores showed normal distribution. The total score of the questionnaire was found to be in accordance with the normal distribution. Since the data conformed to normal distribution, Independent Sample *t* test was used to compare groups of two (gender, special education status, mother-father relationship, parents’ employment status), and One Way ANOVA test was used to compare independent variables containing three or more groups (socioeconomic status, parents’ education levels, family structure). Pearson correlation analysis was used to evaluate the correlation between the scales.

## Results

The age range of the children was 2 to 12 years, with a mean (SD) of 7.63 (3.02) years. Among the children, 88.4% (n: 213) were male. The summarized characteristics of the children are presented in Table 1.

**Table 1:** Children’s characteristics

	n	%	
Gender			
Female	28		11.6
Male	213		88.4
Special education			
No	16		6.6
Yes	225		93.4
Socioeconomic status			
Low	21		8.7
Middle	195		80.9
High	25		10.4
Parents			
Divorced	21		8.7
Married	220		91.3
Family structure			
Nuclear	185		76.8
Extended	37		15.3
Broken (single parent)	19		7.9
Mothers’ education level			
Illiteracy	0		0.0
Primary-secondary school	100		41.5
High school	83		34.4
College or university	58		24.1
Fathers’ education level			
Illiteracy	5		2.1
Primary/secondary school	112		46.5
High school	52		21.6
College or university	72		29.8
Mothers’ work status			
No	188		78.0
Yes	53		22.0
Fathers’ work			
No	29		12.0
Yes	212		88.0
Total	241		100.0

For the analysis aimed at determining construct validity, a Kaiser–Meyer–Olkin value of 0.854 and p-value < .001 were established. Item factor loadings ranged from 0.319 to 0.812, and item-total correlation values ranged from 0.229 to 0.700. The Cronbach alpha coefficient for the questionnaire was 0.921, explaining 38.82% of the total variance. Subscale Cronbach alpha coefficients were 0.813 for “Experience of being a parent,” 0.768 for “Family Life,” 0.810 for “Child Development, Understanding, and Social Relationships,” and 0.804 for “Child Symptoms (Feelings and Behaviour).” The factor loadings of items, corrected item-total correlations, and Cronbach’s alpha coefficients with item removal are presented in Table 2.

**Table 2:** Factor loading, corrected item-total correlations, cronbach alpha coefficients for the items

AFEQ		Factor loading	Corrected item-total correlation	Cronbach alpha coefficient	Cronbach alpha
Experience of being a parent					
1.	Item	0.564	0.443	0.801	0.813
2.	Item	0.515	0.424	0.803	
3.	Item	0.504	0.404	0.804	
4.	Item	0.641	0.519	0.796	
5.	Item	0.696	0.566	0.792	
6.	Item	0.612	0.492	0.798	
7.	Item	0.394	0.298	0.811	
8.	Item	0.638	0.533	0.793	
9.	Item	0.577	0.471	0.798	
10.	Item	0.595	0.480	0.798	
11.	Item	0.511	0.427	0.802	
12.	Item	0.359	0.289	0.817	
13.	Item	0.676	0.584	0.790	
Family life					
14.	Item	0.418	0.317	0.765	0.768
15.	Item	0.504	0.383	0.756	
16.	Item	0.421	0.291	0.766	
17.	*Item*	0.642	0.509	0.736	
18.	Item	0.575	0.419	0.750	
19.	Item	0.734	0.569	0.728	
20.	Item	0.711	0.550	0.735	
21.	Item	0.712	0.566	0.726	
22.	Item	0.594	0.461	0.745	
Child development, understanding and social relationships					
23.	Item	0.456	0.239	0.811	0.810
24.	Item	0.319	0.474	0.795	
25.	Item	0.607	0.576	0.787	
26.	Item	0.705	0.570	0.787	
27.	Item	0.705	0.378	0.802	
28.	Item	0.461	0.602	0.785	
29.	Item	0.706	0.279	0.808	
30.	Item	0.356	0.507	0.792	
31.	Item	0.608	0.454	0.797	
32.	Item	0.567	0.414	0.800	
33.	Item	0.494	0.475	0.795	
34.	Item	0.560	0.390	0.805	
35.	Item	0.472	0.509	0.792	
36.	Item	0.590	0.229	0.814	
Child symptoms					
37.	Item	0.561	0.423	0.793	0.804
38.	Item	0.528	0.384	0.795	
39.	Item	0.410	0.321	0.804	
40.	Item	0.623	0.493	0.785	
41.	Item	0.656	0.502	0.787	
42.	Item	0.723	0.583	0.777	
43.	Item	0.464	0.381	0.799	
44.	Item	0.481	0.409	0.793	
45.	Item	0.498	0.436	0.791	
46.	Item	0.339	0.286	0.804	
47.	Item	0.699	0.574	0.778	
48.	Item	0.812	0.700	0.763	

Cronbach alpha coefficient: 0.921

The four-factor structures established through exploratory factor analysis were assessed for fit using confirmatory factor analysis. The model fit indices indicated an acceptable agreement, with values as follows: χ2/df (2.28), SRMR (0.079), RMSEA (0.073), and PNFI (0.622), indicating a good model fit.

Total scores of the study group ranged from 68 to 216, with a mean (SD) of 130.09 (28.36). Subscale mean scores and total mean scores are provided in Table 3.

**Table 3:** The mean subscale scores

AFEQ	Mean (SD)
Experience of being a parent	30.35 (8.44)
Family life	23.95 (7.04)
Child development, understanding, and social relationships	42.76 (9.72)
Child symptoms	33.03 (9.17)
Total	130.10 (28.36)

*AFEQ* Autism Family Experience Questionnaire

**Fig. 1:**
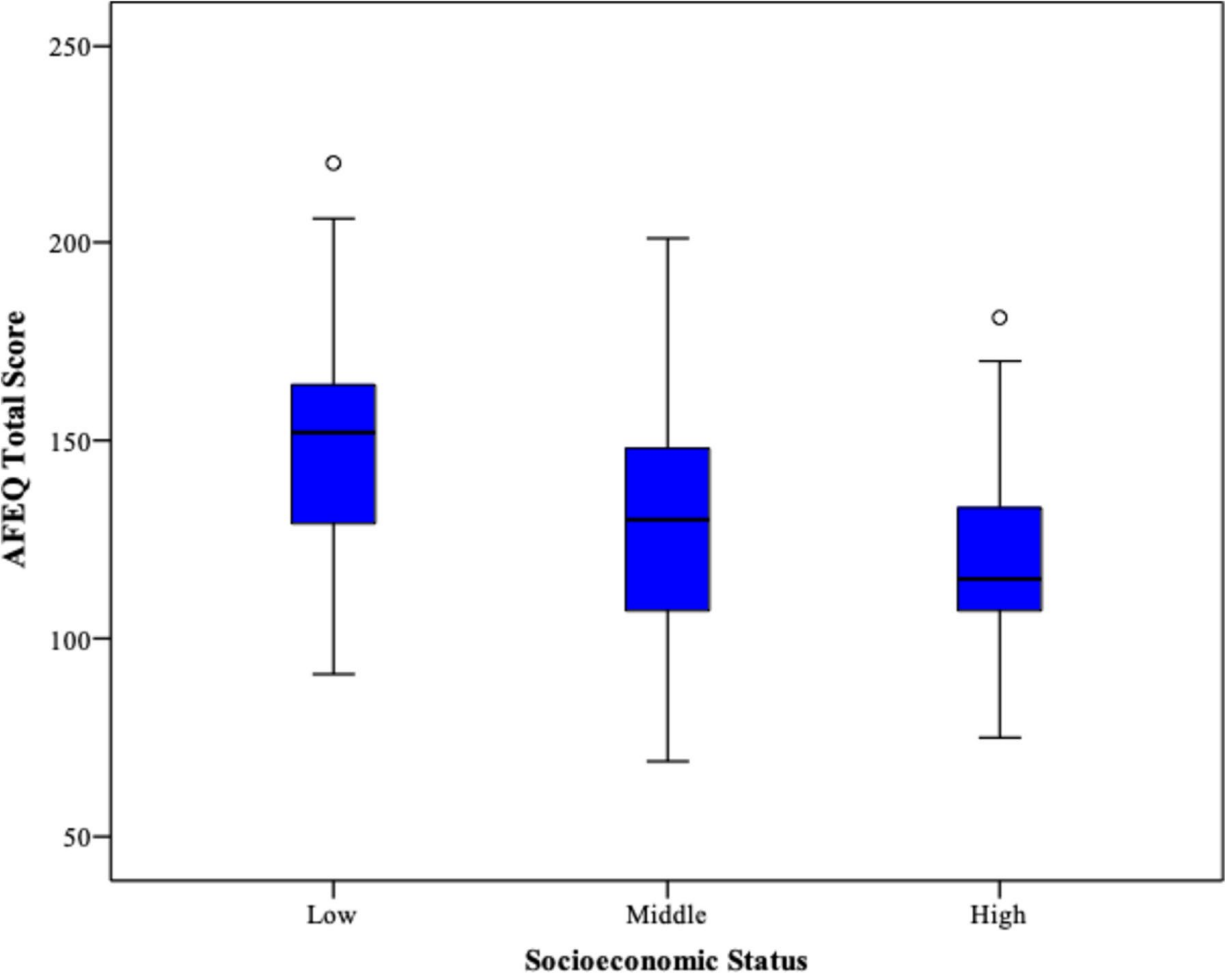
Comparison of the total scores of the groups according to the socioeconomic status

It was found that there were no significant differences in age, gender, parents’ relationship, education level, and family structure variables among the groups when evaluated based on socioeconomic status. However, based on Spearman Correlation analyses, in the"Experience of being a parent” subscale, those with low socioeconomic status had a worse score than those with middle (*p* = .014) and high (*p* = .006). In the subscale of “Child development, understanding, and social relationships”, those with low socioeconomic status also had a worse score than those with middle (*p* = 0.008) and high (*p* = .007). In the subscale of “Child symptoms”, those with low socioeconomic status also had a worse score than those with middle (p: .030). However, there was no significant difference regarding socioeconomic status groups in the “family life” subscales. The comparison of the total scores of the groups according to socioeconomic status is shown in Fig. 1.

The Autism Behavior Checklist total score ranged from 4 to 150, with a mean (SD) of 65.74 (32.38). The Quality of Life (QoL) score ranged from 89 to 217, with a mean (SD) of 138.05 (21.53). Moderate positive correlations were found between AFEQ and the Autism Behavior Checklist (r: 0.555; *p* < .001). Moderate negative correlations were observed between AFEQ and parts A (r: − 0.615; *p* < .001) and B (r: − 0.504; *p* < .001) of the Quality of Life in Autism Questionnaire. The correlation analysis results are detailed in Table 4.

**Table 4:** The results of the correlation analysis between the AFEQ and the ABC scale and the quality of life scale and its subscales

AFEQ	Autism behaviour checklist						QoL		
	Sensory	Relating	Body and object use	Language	Social and self-help	Total ABC	QoL-A	QoL-B	Total QoL
Experience of being parent	r: 0.234*	r: 0.250*	r: 0.236*	r: 0.103**	r: 0.263*	r: 0.263*	r: − 0.543*	r: − 0.289*	r: − 0.505*
Family life	r: 0,347*	r: 0.401*	r: 0.359*	r: 0.246*	r: 0.433*	r: 0.431*	r: − 0.597*	r: − 0.412	r: − 0.608*
Child development, understanding, and social relationships	r: 0.497*	r: 0.550*	r: 0.494*	r: 0.239*	r: 0.468*	r: 0.550*	r: 0–0.476*	r: − 0.505*	r: − 0,585*
Child symptoms	r: 0.474*	r: 0.543*	r: 0.510*	r: 0.252*	r: 0.524*	r: 0.562*	r: − 0.441*	r: − 0.440*	r: − 0.525*
Total	r: 0.479*	r: 0.538*	r: 0.493*	r: 0.255*	r: 0.515*	r: 0.555*	r: − 0.615*	r: − 0.504*	r: − 0.670*

*AFEQ* Autism Family Experience Questionnaire, *ABC* Autism Behaviour Checklist, *QoL* Quailty of Life

∗*p* < .001, ∗∗*p* > .05

## Discussion

This study aimed to assess the reliability and validity of the Turkish version of the Autism Family Experience Questionnaire (AFEQ) among Turkish parents of children with ASD. The AFEQ, comprising 48 Likert-scale items, was designed to comprehensively explore the experiences of parents raising children diagnosed with autism spectrum disorder. It addresses various dimensions including parenting experiences, family life, child development, and emotional-behavioral symptoms.

In our study, 88% of the children who participated were male. According to Zeidan et al. (2022), previous research has shown that the male-female ratio is typically reported to be around 4/1 or 5/1. The gender ratio observed in our study appears to be slightly higher than the ratios found in recent studies. It is believed that this difference may be due to the cross-sectional study method and could be coincidental.

The present study provides evidence that the adapted Turkish version of AFEQ is a valid and reliable instrument for families with children aged 2 to 12 years on the autism spectrum. The internal consistency of AFEQ, as measured by Cronbach’s alpha (α), yielded a value of 0.921, indicating strong reliability. Subscale Cronbach α values were also substantial: experience of being a parent (0.813), family life (0.768), child development, understanding and social relationships (0.810), and child symptoms (0.804). These findings are consistent with the original study conducted by Leadbitter et al. (2018), who reported α values of 0.85 for the parent subscale, 0.83 for family, 0.81 for child development, 0.79 for child symptoms, and 0.92 for the overall AFEQ total. The observed Cronbach α values align closely with the original version.

In terms of model fit, confirmatory factor analysis (CFA) yielded fit indices suggesting a well-fitting model, with values including 2.28 for Chi-square/degrees of freedom, 0.076 for Root Mean Squared Approximation Error (RMSEA), 0.082 for Standardized Root Mean Residual Squares (SRMR), and 0.606 for the Parsimony Normed Fit Index (PNFI). These indices collectively indicate the model’s strong fit, which was also confirmed by comparing with other relevant studies (Hu & Bentler, 1999).

Analysis of total and subscale scores revealed a significant correlation between socioeconomic status and subscale scores related to the experience of being a parent, child development, understanding, and social relationships. Interestingly, parents from families with higher socioeconomic status reported lower scores in the experience of being a parent subscale compared to those from middle and low socioeconomic status families. This may reflect varying stressors and challenges faced by parents across socioeconomic strata. The complex interplay of autism severity, emotional and behavioral issues, language development, and parental characteristics such as mental health, coping mechanisms, and access to support likely contributes to this observation. Parents with higher socioeconomic status may have more positive experiences due to factors such as greater available time, stronger social support networks, financial stability, and easier access to healthcare and education services (Kelly et al., 2019; Pickard et al., 2016).

The literature underscores the significant influence of family financial well-being on the development of children with autism spectrum disorder (ASD) (Parish et al., 2015). Consistent with this, the findings of our study align with studies suggesting a positive correlation between higher socioeconomic status and scores in the child development, understanding, and social relationships subscale. This could be attributed to the advantages that families with better financial standing have in terms of early autism diagnosis and access to intervention programs (Kelly et al., 2019). Leadbitter et al. (2018) demonstrated the contribution to the development and understanding of children in order to evaluate the effectiveness of intervention methods applied to children with autism. At this point, it is not surprising that parents with good financial status have easier access to intervention programs for their children and, as a result, report more progress.

Our results highlight a negative correlation between AFEQ total and subscale scores and the Quality of Life in Autism Questionnaire. Parents of children on the autism spectrum reported lower subjective physical and mental well-being, diminished social functioning, and less satisfaction with social environments compared to parents of typically developing children (Vasilopoulou et al., 2016). The association between greater autism severity, poorer social functioning, internalizing problems, and restricted and repetitive behaviors with decreased quality of life is supported by previous studies (Cappe et al., 2018; Eapen et al., 2022; Sikora et al., 2013). Consistent with the studies showing the relationship between cognitive functions, socialization and quality of life, significant correlations have been found between the child development, understanding, and social relationships subscale of the AFEQ and the quality of life in autism questionnaire in this current study (Renford et al., 2020).

Interestingly, AFEQ total and subscale scores exhibit positive correlations with various subscales and total scores of the Autism Behavior Checklist, specifically those related to sensory, relating, body and object use, language, social, and self-help domains. This aligns with studies demonstrating the relationship between socialization, language skills, and quality of life (Osborne et al., 2010; Baghdadli et al., 2014). Parents’ concerns about their children’s language and communication skills have been linked to lower quality of life (Eapen et al., 2022; Osborne et al., 2010).

The study’s limitations should be acknowledged, including its focus on the Turkish context, which limits direct cross-cultural comparison. Additionally, although the selected sample is representative of the urban population in Turkey in terms of demographic characteristics, potential bias could arise from the sampling method, which collected cases predominantly from urban settings. Future research could expand the sample to include rural populations for a more comprehensive validation.

While the original version of the AFEQ suggested that it reflects changes resulting from intervention programs for autism (Leadbitter et al., 2018), future studies should evaluate the scale within the context of intervention programs in the present study’s setting.

### Implications

In conclusion, the findings of this study demonstrate the cultural appropriateness and validity of the Turkish version of the AFEQ among a Turkish sample. This version can now be more widely applied. The AFEQ not only enables parents to report their individual and family priorities but also offers a comprehensive understanding of emotional and behavioral symptoms in children with autism. It provides insights into child development, understanding, social relations, and parental experiences. This multifaceted view helps us grasp the child holistically within their microenvironment, and better anticipate changes resulting from intervention programs.
